# Fertility Preservation in Pediatric Oncology: Results of a Single-Center Retrospective Study (2000–2018)

**DOI:** 10.3390/cancers17223615

**Published:** 2025-11-10

**Authors:** Jonas Hafele, Gabriele Kropshofer, Roman Crazzolara, Bettina Toth, Bettina Böttcher

**Affiliations:** 1 Department of Pediatrics, Medical University of Innsbruck, Anichstraße 35, 6020 Innsbruck, Austria; jonas.hafele@student.i-med.ac.at (J.H.); gabriele.kropshofer@tirol-kliniken.at (G.K.); roman.crazzolara@tirol-kliniken.at (R.C.); 2 Department of Gynecological Endocrinology and Reproductive Medicine, Medical University of Innsbruck, Anichstraße 35, 6020 Innsbruck, Austria; bettina.toth@i-med.ac.at

**Keywords:** childhood cancer survivors, gonadotoxicity, cryopreservation, survivorship, infertility, FertiPROTEKT e.V.

## Abstract

**Simple Summary:**

Advances in cancer treatment have significantly increased survival rates among children and adolescents, but many therapies may affect future fertility and impact long-term quality of life. Preserving fertility has, therefore, become a key element of pediatric cancer care. This retrospective study analyzed how fertility preservation was managed for young patients treated for cancer at the Medical University of Innsbruck between January 2000 and December 2018. The aim was to better understand current practices, identify gaps, and raise awareness among physicians about possible long-term effects on fertility. As comprehensive international data on the extent to which fertility preservation is offered and performed in these contexts is still lacking, our findings contribute to closing this knowledge gap.

**Abstract:**

**Background/Objectives**: With increasing survival rates in pediatric oncology, late effects, such as therapy-induced infertility, are becoming more relevant. This study evaluated the management of fertility preservation in children and adolescents with cancer at the Medical University Innsbruck between 2000 and 2018. **Methods**: In this retrospective monocentric study, 552 patients (0–17 years) receiving chemotherapy were analyzed. Data was extracted from the Clinical Information System and the cryopreservation database. The assessed main variables included pubertal status, sex hormone levels, and use of fertility preservation methods. **Results**: Fertility preservation was documented in 6.5% of patients, more frequently in males (8.9%) than females (3.2%). Sperm cryopreservation was performed in twenty-eight males, ovarian tissue cryopreservation in six females, and oocyte cryopreservation in three. Pubertal status at diagnosis was recorded in 4.9% of patients and hormone levels in 29.7%. **Conclusions**: The findings highlight significant gaps in systematic fertility preservation, particularly in female patients. Consistent assessment of pubertal and hormonal parameters at diagnosis is essential to inform decision-making. Standardized procedures and closer interdisciplinary collaboration are needed to ensure equitable access to fertility preservation and safeguard long-term quality of life.

## 1. Introduction

With the increasing number of childhood cancer survivors (CCSs), minimizing late effects and improving quality of life (QoL) have become increasingly important. In addition to physical limitations such as organ dysfunction, endocrine deficits, sterility/infertility, or psychosocial stressors also occur [1,2,3,4]. For survivors, fertility impairment is considered one of the most relevant long-term effects, the assessment of which is complicated by heterogeneous study populations and the frequent lack of long-term contact with oncologists [5].

In males, fertility disorders primarily arise from damage to spermatogenesis or the hypothalamic–pituitary–gonadal (HPG) axis. Hypogonadism and erectile dysfunction are other possible consequences [6,7,8]. Studies show a significantly increased risk of infertility in male CCSs [9,10]. In women, the loss of primordial follicles, uterine damage, HPG disorders, and vaginal strictures can cause infertility resulting from chemotherapy or irradiation [11,12]. Female CCSs have a lower pregnancy rate compared to the general population, mainly due to the development of premature ovarian insufficiency (POI) [13,14,15].

Rendtorff et al. reported that around one-third of CCSs experience fertility limitations, although the desire to have children remains comparable to that of the general population [16]. Abortions are less common in CCSs, which underscores the importance of the desire to have children [17].

The degree of fertility impairment depends on the type and dose of therapy, underlying disease, gender, and pubertal status. Chemotherapeutic agents, especially alkylating agents and platinum compounds, are considered gonadotoxic and can cause long-term fertility impairment [18,19,20]. Men show dose-dependent disorders of spermatogenesis, including azoospermia, while women show a decrease in follicular reserve and an increased risk of POI [21,22,23,24]. Platinum compounds, especially cisplatin, also affect fertility in both sexes, although the data is heterogeneous [25,26,27,28].

Radiotherapy can impair fertility both through direct gonadal damage and through central effects on the HPG axis. Pelvic and whole-body irradiation in particular increase the risk of ovarian insufficiency, uterine dysfunction, or damage to spermatogenesis [10,14,16,28,29,30,31]. The results for cranial irradiation are contradictory, especially in male CCSs [10,14,16,32,33].

Puberty status influences vulnerability to gonadotoxic therapies. Prepubertal girls appear to be more resistant to certain chemotherapies, while post-pubertal girls show a higher risk of fertility disorders, whereas in boys, the degree of impairment does not seem to correlate with the stage of puberty [16,28,34].

Since there is no safe threshold above which infertility occurs after cancer therapy, all patients should be informed about the risks and offered fertility protection options depending on their gender, age, underlying disease, and therapy [35].

The options for fertility preservation in prepubertal boys are limited. In addition to testicular shielding during radiotherapy, testicular tissue cryopreservation (TTC) is being tested experimentally. Successful sperm maturation from stem cells has not yet been achieved in humans but is already possible in animal models [36,37,38].

Once spermarche has occurred, sperm cryopreservation (SC) can usually be performed in post-pubertal boys. Alternatively, in rare cases, penile vibratory stimulation (PVS), electrostimulation, or testicular sperm extraction (TESE) can also be used if necessary [35,39,40]. Cryopreservation of testicular tissue is not considered a standard procedure but experimental. It is important to obtain sperm before starting therapy, as sperm quality declines after therapy begins. Hormonal protective measures are not effective and are not recommended by professional societies [35,40].

The most promising option for prepubertal girls is ovarian tissue cryopreservation (OTC), which has already enabled puberty induction and live births after re-transplantation [41,42,43], although the risk of malignant cell contamination must be considered. Ovarian transposition or gonadal shielding may also be considered [35,44,45,46].

In addition, oocyte cryopreservation (OC) after controlled ovarian hyperstimulation (COH) and the use of gonadotropin-releasing hormone agonists (GnRHas) are applied procedures in post-pubertal girls, although the latter is not established. The administration of GnRHas is contradictory but can be offered as a complementary option [35,40,47,48]. OC achieves pregnancy rates of up to 50% in young women [49,50] but requires almost two weeks of preparation time. OTC is increasingly considered a standard procedure, although its invasiveness and higher resource requirements should be taken into account [40].

This study aimed to evaluate the current approach to fertility preservation for pediatric cancer patients at the Department of Pediatrics in Innsbruck between 2000 and 2018. The focus was on documenting the services offered and how they were implemented prior to the start of therapy, analyzing existing practices, and identifying potential care gaps, with the aim of improving education about the long-term effects of gonadotoxicity.

## 2. Materials and Methods

This retrospective, monocentric study included 552 pediatric patients (305 male, 247 female) aged 0–17 years treated for oncological diseases at the Department of Pediatrics I, Medical University of Innsbruck, between 2000 and 2018. All oncological patients receiving chemotherapy were included; those without such treatment or with incomplete records were excluded. The study period was limited to 2000–2018 to allow the use of an existing, comprehensive dataset of pediatric oncology patients who received chemotherapy, which served as the foundation for the systematic collection and analysis of additional clinical information by the authors. The network FertiPROTEKT e.V. was founded in German-speaking countries in 2006. Data of all girls and female adolescents were also documented in the database provided by the network, but for the current study, data for the whole period of time, as well for, males and females were collected from the Clinical Information System and the cryopreservation database of the Department of Gynecological Endocrinology and Reproductive Medicine and the Department of Pediatrics of the Medical University of Innsbruck. The Department of Gynecological Endocrinology and Reproductive established biobanking of gametes and ovarian tissue in the year 2003. The data was pseudonymized via consecutive study numbers.

The parameters included demographics, diagnosis, treatment details, pubertal stage, hormone levels (FSH, AMH, estradiol, testosterone), menarche and spermarche age, and fertility preservation measures (SC, OTC, OC). Descriptive statistics were applied using IBM SPSS Statistics 26 and Microsoft Excel 365.

The study was approved by the local Ethics Committee of the Medical University of Innsbruck (1374/2023). The corresponding author will provide data upon reasonable request.

## 3. Results

### 3.1. Study Population

From 2000 to 2018, a total of 552 pediatric patients (306 males [55.4%], 246 females [44.6%]) aged 0–17 years received chemotherapy for oncological diseases at the Department of Pediatrics I, Medical University of Innsbruck. According to the average age of the beginning of puberty [51,52], patients were analyzed in one prepubertal group below the age of 12 and one group over the age of 12. At the time of data collection, 20.1% of patients were deceased. The most frequent diagnosis was acute lymphoblastic leukemia (ALL, *n* = 199), followed by extracranial solid tumors (*n* = 166, mainly neuroblastoma, Ewing sarcoma, osteosarcoma, rhabdomyosarcoma, and Wilms tumor), central nervous system (CNS) tumors (*n* = 63), non- Hodgkin lymphoma (NHL, *n* = 37), acute myeloid leukemia (AML, *n* = 35), Hodgkin lymphoma (*n* = 32), and rare pediatric tumors (*n* = 20). At least one recurrence occurred in 22.5% of patients, and 29.3% received radiotherapy, including cranial (*n* = 57) and extracranial (*n* = 105) irradiation (see Table 1).

ThSPSSe median age at diagnosis was 6 years (IQR = 2–12). The highest frequency of diagnoses was observed in children aged 0–4 years (*n* = 240, 43.5%). The mean number of new oncological diagnoses that met the inclusion criteria was 29 per year (range: 16–38). Extracranial solid tumors, AML, CNS tumors, and rare pediatric malignancies peaked in infancy and early childhood, while the incidence of ALL was highest between ages 2 and 4. NHL manifested at a consistent frequency across all age groups, while Hodgkin lymphoma was rarely diagnosed before the age of 10, and its incidence increased thereafter.

### 3.2. Indicators of Puberty

The pubertal stage was documented in 27 patients (4.9%). Among the girls (*n* = 9), the development of breast and pubic hair was documented. The age or date of menarche was recorded in *n* = 44 patients. In 10 of these girls, menarche occurred before the date of initial diagnosis. An evaluation of testicular development and pubic hair was conducted among a total of *n* = 18 males. The age of the patients, for whom puberty indicators were noted, ranged from 5 to 17 years (M = 11.7). Hormone levels were measured in 164 patients (29.7%), including FSH, estradiol, AMH, and testosterone. Evidence of hormonal activation consistent with puberty was observed in 30.6% of females and 20.2% of males, where sex hormones were documented (see Table 2).

### 3.3. Fertility Preservation

The number of counseling sessions about fertility preservation cannot be analyzed due to missing data in documentation in the individual medical records, but the analysis revealed that fertility-preserving interventions were performed in 6.5% of all included patients (*n* = 36). Among patients aged ≥12 years, 25.4% (36/142) received a fertility preservation, with higher rates in males (31.8%) than females (14.8%). In the male subject group, twenty-seven underwent sperm cryopreservation by masturbation, while one boy underwent TESE. Cryopreservation of testicular tissue, which is still considered experimental, has not been offered to boys at all, as no study protocol to examine the procedure was established at that time. Among the females, six underwent ovarian tissue cryopreservation, while three underwent oocyte cryopreservation. In one case, both OTC and OC were performed in the same patient (see Table 3). In this period, all oocyte retrievals as well as cryopreservation of ovarian tissue took place before the initiation of chemotherapy.

**Figure 1:**
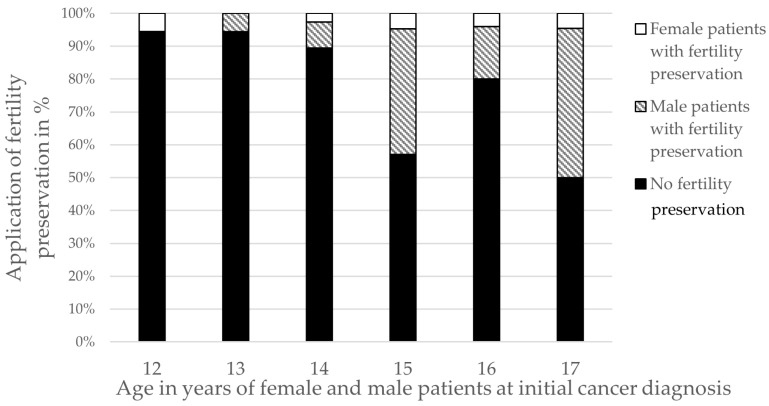
Application of fertility preservation measures in male and female patients as a percentage of the total age group, starting from the age of 12 years to the age of 17 years at initial cancer diagnosis.

The mean age at fertility preservation was 15.7 years for males (range 13–17) and 14.8 years for females (range 12–17). The proportion of interventions increased with age, reaching 50% among 17-year-old patients, with a male-to-female ratio of 10:1 (see Figure 1). No procedures were performed in patients under 12 years.

**Figure 2:**
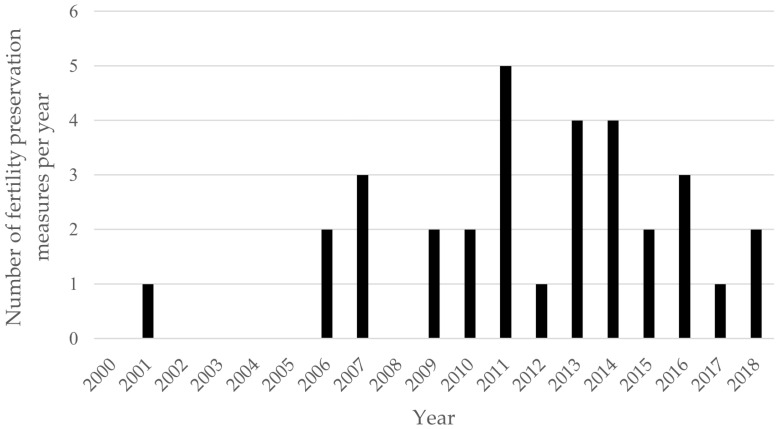
Number of fertility preservation measures from 2000 to 2018 in pediatric patients at one single center per year.

The highest frequency of interventions was observed in patients with extracranial solid tumors (*n* = 14, mainly Ewing sarcoma and osteosarcoma) and ALL (*n* = 8). Fertility preservation procedures were initiated in 2006, with a single exception in 2001. The mean number of interventions per year since 2006 was 2.4, with a maximum of five recorded in 2011 (see Figure 2).

**Figure 3:**
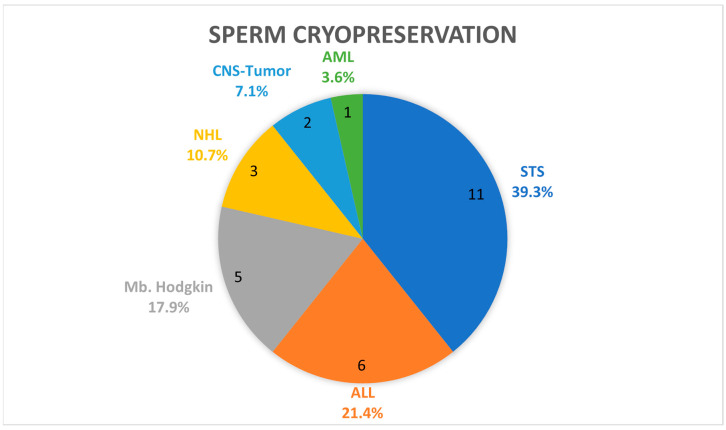
Sperm cryopreservation in boys stratified by diagnosis; *n* = 28; NHL = non-Hodgkin lymphoma; CNS = central nervous system; AML = acute myeloid leukemia; STS = soft tissue sarcoma; M. Hodgkin = Hodgkin lymphoma.

When fertility preservation measures were stratified by diagnosis, it was shown that oocyte cryopreservation was mostly performed in girls with Ewing sarcoma, rhabdomyosarcoma, and Hodgkin lymphoma. Cryopreservation of ovarian tissue was performed in one third of the patients presenting with soft tissue sarcoma, one third with acute lymphocytic leukemia, and one third with Hodgkin lymphoma. Diagnoses of boys with sperm cryopreservation are shown in Figure 3.

This retrospective study covered a period of almost 20 years in a pediatric population. Over 20% of the patients deceased. We encountered a considerable amount of missing data. The status of puberty was missing in 95% and the documentation of fertility preservation was missing in 93%, whereas hormone levels were missing in around 70% of all girls and boys.

## 4. Discussion

This monocentric, retrospective study assessed fertility preservation practices in pediatric oncology patients treated in Innsbruck between 2000 and 2018. The documentation of puberty status, hormone levels, and reproductive milestones (menarche and spermarche) was inconsistent, particularly in the early study period. This finding suggests that there was historically limited awareness of fertility risks as well as a lack of interventions to preserve fertility. The essential first step is the awareness of the specialists responsible for the primary treatment of patients. They need to address possible fertility impairment and enable patients to receive counselling. For the patient, this additional aspect opens up a new field of problems and, at the time of cancer diagnosis, introduces another complex area of concern. In children and adolescents, the desire to have children and to build a family is far beyond the horizon.

Existential fears of pain, suffering, and death, as well as the prognosis of the disease, are central to the parent’s and, depending on the age and intellectual maturity, the patient’s experience [53]. During medical consultations, physicians usually focus on explaining the diagnosis and therapeutic options, whereas the effects of the disease and its treatment on fertility are not always an integral part of counseling [54]. Nevertheless, patients express a clear need to receive information on these topics [55]. Nearly 60% of parents of children with cancer reported concern about their child’s potential infertility [56]. Nevertheless, the proportion of patients receiving counseling on fertility-preserving measures remains relatively low. Only 34% of premenopausal breast cancer patients recalled having received counseling about fertility and menopause [57]. In another survey, 72% of breast cancer patients reported receiving information about potential impacts on fertility, but only 17% had been counseled by a fertility specialist [54]. Even among men—despite the relative simplicity of sperm cryopreservation—only 51–57% reported being informed about this option [58]. Although counseling about fertility preservation has been increasingly taken into account by treating physicians over the last 20 years, many women and men still report feeling insufficiently informed [59]. During individualized counseling on fertility-preserving measures, an interdisciplinary consensus should consider factors, like planned therapy, overall prognosis, individual ovarian reserve, potential risks, and contraindications of a future pregnancy, as well as the patient’s personal wishes and expectations.

Especially, the risk of infertility needs to be assessed despite the fact that data, especially of recently introduced treatment plans and medications, like checkpoint inhibitors or antibodies, is scarce. Recent study protocols aim to assess this risk in order to improve counseling and to address the patients at risk [60]. Although patients need to know about fertility preservation, overtreatment needs to be avoided if the risk for future infertility is low. Furthermore, autonomous decision-making does not mean that the patient decides to utilize these measures. It could also mean deciding against taking these measures due to different reasons like cost, time, focus on the treatment, or no desire to build a family. In Austria, all measures of fertility preservation are not covered by the public health system.

In children or young adolescents, this decision becomes even more complex [59]. According to the recommendations of the American Society of Clinical Oncology (ASCO), fertility preservation should also be discussed with children; however, there is no consensus regarding a minimum age for such discussions [40,61]. Parents are involved in the decision-making process in order to act in the best interest of the child when the girl or boy is too young to make an autonomous decision [62]. By providing fertility preservation measures, the girl or boy may later, as an adult, decide for herself/himself whether to make use of these preserved materials. The right to parenthood should not be denied to children [63]. In this way, reproductive autonomy is deferred to a later stage of life [64]. Our own data in the subgroup analysis of the same cohort of girls and female patients showed that only a small number used cryopreserved embryos to achieve a pregnancy, as even a higher percentage of patients conceived naturally [65].

These considerations are influenced by moral and religious beliefs as well as the life concepts of the parents. In clinical practice, once a certain level of understanding and insight is reached—typically during puberty—decision-making competence rests with the adolescent [66]. Interventions cannot be carried out against the patient’s will; therefore, while parental arguments must be respected, they should not be the decisive factor in the final decision. Parental decisions regarding fertility preservation are permissible provided the child agrees and the intervention is expected to confer a benefit to the child. It is considered an ethical obligation to decide in the child’s best interest when fertility preservation measures are contemplated [67]. Both parents and children should receive appropriate information under the principle of informed consent. The initiation of oncologic therapy should not be delayed, and the child’s quality of life should not be substantially compromised.

Our analysis revealed that 6.5% of patients underwent procedures of fertility preservation, with a significantly higher uptake among males (8.9%) compared to females (3.2%). Among patients aged ≥12 years, 25.4% received such interventions, a proportion that was again higher among male patients (31.8%) than among female patients (14.8%). This gender imbalance is consistent with reports from other European centers, where SC is more accessible and logistically feasible than OTC or OC [68,69]. The invasiveness, prolonged preparation time, and elevated resource requirements (including specialized staff, facilities, and associated costs) of female procedures are likely to explain the observed differences. It must be noted that these interventions are not covered by public insurance in Austria. Consequently, the financial burden of these treatments falls on the families of patients, which may further impact the number of patients undergoing preservation interventions. This provides an additional explanation for the gender imbalance, as measures for girls/women cost ten times as much as for males. Furthermore, most diagnoses, particularly ALL and AML, require immediate treatment, often started within hours of diagnosis, making any delay for fertility preservation procedures challenging. At present, the safety and practicality of such procedures remain uncertain, as leukemic cells may infiltrate vital organs, including the ovaries and testes.

International data on fertility preservation in pediatric oncology is sparse and heterogeneous, which limits the comparability of the results. A Swiss survey conducted from 2009 to 2013 reported a 9% uptake [68], which is slightly higher than the rate observed in our cohort. However, it should be noted that the inclusion criteria differed between the two studies, as they also included non-malignant diseases requiring HSCT as well as patients treated with radiotherapy alone. A European multicenter survey revealed significant variability, with sperm cryopreservation being offered in the majority of centers that supplied their data, while oocyte preservation was available in only one-third of the centers [69]. Beyond these examples, few systematic publications exist, highlighting the unique relevance of our findings. Data on actual uptake and documentation practices is rarely reported and urgently needed to inform guidelines and improve equity in care.

It is important to acknowledge the limitations of the study. The single-center, retrospective design of the study limited its generalizability, and missing data (e.g., Tanner staging, hormone values) restricted the analysis. No risk stratification has been performed. The results may be influenced by physician-dependent variation in awareness and counseling, as no standard operating procedures (SOPs) were in place during the study period. Despite these limitations, the dataset provides valuable insights into historical practice and offers a rare reference point for international comparison.

### Practical Implications

Our findings underline the need for structured, prospective approaches. Systematic documentation of puberty status, hormone levels (including AMH in girls and inhibin B in boys), and reproductive milestones should be standard at diagnosis. At our clinic, as a result of this study, an SOP was implemented. Puberty status, menarche, hormonal evaluation, and an infertility risk stratification system within an interdisciplinary approach involving pediatric oncologists and reproductive specialists has been established.

Awareness among health care professionals, patients, and families should be increased through standardized information at diagnosis. Fertility preservation should eventually be re-evaluated at a later stage of therapy, for example, prior to stem cell transplantation. Finally, expanding fertility-preserving options for high-risk patients, including experimental approaches, should be considered to prepare for future technological advances.

## 5. Conclusions

Fertility preservation in pediatric oncology patients at our center was inconsistently applied, with marked gender differences and incomplete documentation of reproductive parameters. Although uptake rates were comparable to the few international reports available, the scarcity of published data underscores the particular relevance of our findings.

Standardized documentation, interdisciplinary decision-making, and broader access to fertility preservation are essential to optimize care. As survival after childhood cancer improves, fertility preservation must become an integral component of survivorship care to safeguard long-term quality of life and future family planning.

## Figures and Tables

**Table 1:** Patient characteristics, survival, recurrence, and radiotherapy status.

Variable	*n*	Percentage (%) ^1^	<12 Years *n* (%)	>12 Years *n* (%)
Sex				
Female	246	44.6	192 (78.0)	54 (22.0)
Male	306	55.4	218 (71.2)	88 (28.8)
Survival, recurrence, and radiation status				
Alive	441	79.9	f: 153 m: 176	f: 43 m: 69
Deceased	111	20.1	f: 39 m: 42	f: 11 m: 19
Recurrence (min. 1)	124	22.5	f: 43 m: 43	f: 15 m: 23
Radiation	162	29.3	f: 48 m: 55	f: 21 m: 38
Extracranial radiation	105	19.0	f: 33 m: 30	f: 17 m: 25
Cranial radiation	57	10.3	f: 15 m: 25	f: 4 m: 13

^1^ Percentage of the total sample; f = female; m = male.

**Table 2:** Clinical documentation of sex hormone status.

Variable	*n*	Percentage (%) ^1^
Total (female)	75	30.4
AMH	4	1.6
FSH	73	29.6
Estradiol	72	29.1
Hormonally active	23	30.6 ^2^
Total (male)	89	29.2
FSH	88	28.9
Testosterone	82	26.9
Hormonally active	18	20.2 ^2^

^1^ Percentage of the total sample of the same sex; ^2^ percentage of the total number of patients with documented hormone levels of the same sex in female patients with estradiol ≥ 25 ng/L and age ≥ 9 years and in male patients with testosterone ≥ 3.1 µg/L and age ≥ 9 years.

**Table 3:** Application of sperm cryopreservation (SC), oocyte cryopreservation (OC), or ovarian tissue cryopreservation (OTC).

Variable	*n*	Percentage (%) ^1^
Fertility preservation (female)		
No	239	96.8
Yes	8	3.2
OTC *	6	2.4
OC *	3	1.2
Fertility preservation (male)		
No	277	90.8
Yes	28	9.2
SC via masturbation	27	8.9
SC via TESE	1	0.3

^1^ Percentage of the total sample of the same sex; * one patient underwent OTC and OC.

## Data Availability

The datasets generated and analyzed during the current study are not publicly available due to privacy and ethical restrictions. In accordance with institutional and national data protection regulations, individual-level patient data cannot be shared in an open repository. However, de-identified data may be made available from the corresponding author upon reasonable request and after review of compliance with applicable ethical and legal standards.

## Abbreviations

The following abbreviations are used in this manuscript:

AMH	Anti-Müllerian Hormone
CCS	Childhood Cancer Survivor
COH	Controlled Ovarian Hyperstimulation
HPG	Hypothalamic–Pituitary–Gonadal Axis
HSCT	Hematopoietic Stem Cell Transplantation
NHL	Non-Hodgkin Lymphoma
OC	Oocyte Cryopreservation
OTC	Ovarian Tissue Cryopreservation
PVS	Penile Vibratory Stimulation
POI	Premature Ovarian Insufficiency
QoL	Quality of Life
SC	Sperm Cryopreservation
TESE	Testicular Sperm Extraction
TTC	Testicular Tissue Cryopreservation

## References

[1] Calaminus G., Langer T., Willich N., Beck J.D. (2000). Lebensqualität Und Spätfolgen Bei Kindern Und Jugendlichen Mit Krebserkrankungen. Onkologe.

[2] Phillips S.M., Padgett L.S., Leisenring W.M., Stratton K.K., Bishop K., Krull K.R., Alfano C.M., Gibson T.M., De Moor J.S., Hartigan D.B. (2015). Survivors of Childhood Cancer in the United States: Prevalence and Burden of Morbidity. Cancer Epidemiol. Biomarkers Prev..

[3] Bitsko M.J., Cohen D., Dillon R., Harvey J., Krull K., Klosky J.L. (2016). Psychosocial Late Effects in Pediatric Cancer Survivors: A Report From the Children’s Oncology Group. Pediatr. Blood Cancer.

[4] Zebrack B.J., Chesler M.A. (2002). Quality of Life in Childhood Cancer Survivors. Psychooncology.

[5] Meacham L.R., Burns K., Orwig K.E., Levine J. (2020). Standardizing Risk Assessment for Treatment-Related Gonadal Insufficiency and Infertility in Childhood Adolescent and Young Adult Cancer: The Pediatric Initiative Network Risk Stratification System. J. Adolesc. Young Adult Oncol..

[6] Green D.M., Liu W., Kutteh W.H., Ke R.W., Shelton K.C., Sklar C.A., Chemaitilly W., Pui C.H., Klosky J.L., Spunt S.L. (2014). Cumulative Alkylating Agent Exposure and Semen Parameters in Adult Survivors of Childhood Cancer: A Report from the St Jude Lifetime Cohort Study. Lancet Oncol..

[7] Ritenour C.W.M., Seidel K.D., Leisenring W., Mertens A.C., Wasilewski-Masker K., Shnorhavorian M., Sklar C.A., Whitton J.A., Stovall M., Constine L.S. (2016). Erectile Dysfunction in Male Survivors of Childhood Cancer-A Report from the Childhood Cancer Survivor Study. J. Sex. Med..

[8] Romerius P., Ståhl O., Moëll C., Relander T., Cavallin-Ståhl E., Wiebe T., Giwercman Y.L., Giwercman A. (2009). Hypogonadism Risk in Men Treated for Childhood Cancer. Clin. Endocrinol. Metab..

[9] Claessens J.J.M., Penson A., Bronkhorst E.M., Kremer L.C.M., van Dulmen-den Broeder E., van der Heiden-van der Loo M., Tissing W.J.E., van der Pal H.J.H., Blijlevens N.M.A., van den Heuvel-Eibrink M.M. (2023). Desire for Children among Male Survivors of Childhood Cancer: A DCCSS LATER Study. Cancer.

[10] Green D.M., Kawashima T., Stovall M., Leisenring W., Sklar C.A., Mertens A.C., Donaldson S.S., Byrne J., Robison L.L. (2010). Fertility of Male Survivors of Childhood Cancer: A Report from the Childhood Cancer Survivor Study. J. Clin. Oncol..

[11] Green D.M., Sklar C.A., Boice J.D., Mulvihill J.J., Whitton J.A., Stovall M., Yasui Y. (2009). Ovarian Failure and Reproductive Outcomes after Childhood Cancer Treatment: Results from the Childhood Cancer Survivor Study. J. Clin. Oncol..

[12] Bath L.E., Critchley H.O.D., Chambers S.E., Anderson R.A., Kelnar C.J.H., Wallace W.H.B. (1999). Ovarian and Uterine Characteristics after Total Body Irradiation in Childhood and Adolescence: Response to Sex Steroid Replacement. BJOG Int. J. Obstet. Gynaecol..

[13] Torella M., Riemma G., De Franciscis P., Verde M.L., Colacurci N. (2021). Serum Anti-Müllerian Hormone Levels and Risk of Premature Ovarian Insufficiency in Female Childhood Cancer Survivors: Systematic Review and Network Meta-Analysis. Cancers.

[14] Green D.M., Kawashima T., Stovall M., Leisenring W., Sklar C.A., Mertens A.C., Donaldson S.S., Byrne J., Robison L.L. (2009). Fertility of Female Survivors of Childhood Cancer: A Report from the Childhood Cancer Survivor Study. J. Clin. Oncol..

[15] Anderson R.A., Brewster D.H., Wood R., Nowell S., Fischbacher C., Kelsey T.W., Wallace W.H.B. (2018). The Impact of Cancer on Subsequent Chance of Pregnancy: A Populationbased Analysis. Hum. Reprod..

[16] Rendtorff R., Hohmann C., Reinmuth S., Müller A., Dittrich R., Beyer M., Wickmann L., Keil T., Henze G., Borgmann-Staudt A. (2010). Hormone and Sperm Analyses after Chemo- and Radiotherapy in Childhood and Adolescence. Klin. Padiatr..

[17] Hohmann C., Borgmann A., Keil T. (2011). Re: Induced Abortions in Danish Cancer Survivors: A Population-Based Cohort Study. J. Natl. Cancer Inst..

[18] Donnez J., Martinez-Madrid B., Jadoul P., Van Langendonckt A., Demylle D., Dolmans M.M. (2006). Ovarian Tissue Cryopreservation and Transplantation: A Review. Hum. Reprod. Update.

[19] Bedoschi G., Navarro P.A., Oktay K. (2016). Chemotherapy-Induced Damage to Ovary: Mechanisms and Clinical Impact. Future Oncol..

[20] Meistrich M. (2009). Male Gonadal Toxicity. Pediatr. Blood Cancer.

[21] Sonigo C., Beau I., Binart N., Grynberg M. (2019). The Impact of Chemotherapy on the Ovaries: Molecular Aspects and the Prevention of Ovarian Damage. Int. J. Mol. Sci..

[22] Soleimani R., Heytens E., Darzynkiewicz Z., Oktay K. (2011). Mechanisms of Chemotherapy-Induced Human Ovarian Aging Double Strand DNA Breaks and Microvascular Compromise. Aging.

[23] Poganitsch-Korhonen M., Masliukaite I., Nurmio M., Lähteenmäki P., Van Wely M., Van Pelt A.M.M., Jahnukainen K., Stukenborg J.B. (2017). Decreased Spermatogonial Quantity in Prepubertal Boys with Leukaemia Treated with Alkylating Agents. Leukemia.

[24] Ginsberg J.P. (2011). New Advances in Fertility Preservation for Pediatric Cancer Patients. Curr. Opin. Pediatr..

[25] Tharmalingam M.D., Matilionyte G., Wallace W.H.B., Stukenborg J.B., Jahnukainen K., Oliver E., Goriely A., Lane S., Guo J., Cairns B. (2020). Cisplatin and Carboplatin Result in Similar Gonadotoxicity in Immature Human Testis with Implications for Fertility Preservation in Childhood Cancer. BMC Med..

[26] Zhou B., Kwan B., Desai M.J., Nalawade V., Henk J., Viravalli N., Murphy J.D., Nathan P.C., Ruddy K.J., Shliakhtsitsava K. (2024). Association of Platinum-Based Chemotherapy with Live Birth and Infertility in Female Survivors of Adolescent and Young Adult Cancer. Fertil. Steril..

[27] Chow E.J., Stratton K.L., Leisenring W.M., Oeffinger K.C., Sklar C.A., Donaldson S.S., Ginsberg J.P., Kenney L.B., Levine J.M., Robison L.L. (2016). Pregnancy after Chemotherapy in Male and Female Survivors of Childhood Cancer Treated between 1970 and 1999: A Report from the Childhood Cancer Survivor Study Cohort. Lancet Oncol..

[28] Reinmuth S., Hohmann C., Rendtorff R., Balcerek M., Holzhausen S., Müller A., Henze G., Keil T., Borgmann-Staudt A. (2013). Impact of Chemotherapy and Radiotherapy in Childhood on Fertility in Adulthood: The FeCt—Survey of Childhood Cancer Survivors in Germany. J. Cancer Res. Clin. Oncol..

[29] Wallace W.H.B., Thomson A.B., Kelsey T.W. (2003). The Radiosensitivity of the Human Oocyte. Hum. Reprod..

[30] Larsen E.C., Schmiegelow K., Rechnitzer C., Loft A., Müller J., Andersen A.N. (2004). Radiotherapy at a Young Age Reduces Uterine Volume of Childhood Cancer Survivors. Acta Obstet. Gynecol. Scand..

[31] De Felice F., Marchetti C., Marampon F., Cascialli G., Muzii L., Tombolini V. (2019). Radiation Effects on Male Fertility. Andrology.

[32] Koustenis E., Pfitzer C., Balcerek M., Reinmuth S., Zynda A., Stromberger C., Hohmann C., Keil T., Borgmann-Staudt A. (2013). Impact of Cranial Irradiation and Brain Tumor Location on Fertility: A Survey. Klin. Padiatr..

[33] Wasilewski-Masker K., Seidel K.D., Leisenring W., Mertens A.C., Shnorhavorian M., Ritenour C.W., Stovall M., Green D.M., Sklar C.A., Armstrong G.T. (2014). Male Infertility in Long-Term Survivors of Pediatric Cancer: A Report from the Childhood Cancer Survivor Study. J. Cancer Surviv..

[34] Thomas-Teinturier C., El Fayech C., Oberlin O., Pacquement H., Haddy N., Labbé M., Veres C., Guibout C., Diallo I., De Vathaire F. (2013). Age at Menopause and Its Influencing Factors in a Cohort of Survivors of Childhood Cancer: Earlier but Rarely Premature. Hum. Reprod..

[35] Lambertini M., Peccatori F.A., Demeestere I., Amant F., Wyns C., Stukenborg J.B., Paluch-Shimon S., Halaska M.J., Uzan C., Meissner J. (2020). Fertility Preservation and Post-Treatment Pregnancies in Post-Pubertal Cancer Patients: ESMO Clinical Practice Guidelines. Ann. Oncol..

[36] Fayomi A.P., Peters K., Sukhwani M., Valli-Pulaski H., Shetty G., Meistrich M.L., Houser L., Robertson N., Roberts V., Ramsey C. (2019). Autologous Grafting of Cryopreserved Prepubertal Rhesus Testis Produces Sperm and Offspring. Science.

[37] Goossens E., Jahnukainen K., Mitchell R.T., Van Pelt A.M.M., Pennings G., Rives N., Poels J., Wyns C., Lane S., Rodriguez-Wallberg K.A. (2020). Fertility Preservation in Boys: Recent Developments and New Insights. Hum. Reprod. Open.

[38] Gül Siraz Ü., Hatipoğlu N. (2021). Fertility Preservation Methods in Childhood and Adolescence Cancers: A Review. J. Pediatr. Acad..

[39] Bahadur G., Ling K.L.E., Hart R., Ralph D., Riley V., Wafa R., Ashraf A., Jaman N., Oyede A.W. (2002). Semen Production in Adolescent Cancer Patients. Hum. Reprod..

[40] Oktay K., Harvey B.E., Partridge A.H., Quinn G.P., Reinecke J., Taylor H.S., Wallace W.H., Wang E.T., Loren A.W. (2018). Fertility Preservation in Patients with Cancer: ASCO Clinical Practice Guideline Update. J. Clin. Oncol..

[41] Poirot C., Abirached F., Prades M., Coussieu C., Bernaudin F., Piver P. (2012). Induction of Puberty by Autograft of Cryopreserved Ovarian Tissue. Lancet.

[42] Matthews S., Picton H., Ernst E., Andersen C. (2018). Successful Pregnancy in a Woman Previously Suffering from β-Thalassemia Following Transplantation of Ovarian Tissue Cryopreserved before Puberty. Minerva Ginecol..

[43] Demeestere I., Simon P., Dedeken L., Moffa F., Tsépélidis S., Brachet C., Delbaere A., Devreker F., Ferster A. (2015). Live Birth after Autograft of Ovarian Tissue Cryopreserved during Childhood. Hum. Reprod..

[44] Balcerek M., von Wolff M., Borgmann-Staudt A. (2020). Kinderonkologische Erkrankungen. Indikation und Durchführung Fertilitätsprotektiver Maßnahmen bei Onkologischen und Nicht-Onkologischen Erkrankungen.

[45] Mossa B., Schimberni M., Di Benedetto L., Mossa S., Schimberni M. (2015). Ovarian Transposition in Young Women and Fertility Sparing. Eur. Rev. Med. Pharmacol. Sci..

[46] Irtan S., Orbach D., Helfre S., Sarnacki S. (2013). Ovarian Transposition in Prepubescent and Adolescent Girls with Cancer. Lancet Oncol..

[47] Dolmans M.M., Taylor H.S., Rodriguez-Wallberg K.A., Blumenfeld Z., Lambertini M., von Wolff M., Donnez J. (2020). Utility of Gonadotropin-Releasing Hormone Agonists for Fertility Preservation in Women Receiving Chemotherapy: Pros and Cons. Fertil. Steril..

[48] Blumenfeld Z. (2019). Fertility Preservation Using GnRH Agonists: Rationale, Possible Mechanisms, and Explanation of Controversy. Clin. Med. Insights Reprod. Health.

[49] Cobo A., García-Velasco J.A., Coello A., Domingo J., Pellicer A., Remohí J. (2016). Oocyte Vitrification as an Efficient Option for Elective Fertility Preservation. Fertil. Steril..

[50] von Wolff M., Bruckner T., Strowitzki T., Germeyer A. (2018). Fertility Preservation: Ovarian Response to Freeze Oocytes Is Not Affected by Different Malignant Diseases—An Analysis of 992 Stimulations. J. Assist. Reprod. Genet..

[51] Largo R.H., Prader A. (1983). Pubertal development in Swiss boys. Helv. Paediatr. Acta.

[52] Largo R.H., Prader A. (1983). Pubertal development in Swiss girls. Helv. Paediatr. Acta.

[53] Tschudin S., Bitzer J. (2009). Psychological aspects of fertility preservation in men and women affected by cancer and other life-threatening diseases. Hum. Reprod. Update.

[54] Partridge A.H., Gelber S., Peppercorn J., Sampson E., Knudsen K., Laufer M., Rosenberg R., Przypyszny M., Rein A., Winer E.P. (2004). Web-based survey of fertility issues in young women with breast cancer. J. Clin. Oncol..

[55] Thewes B., Meiser B., Taylor A., Phillips K.A., Pendlebury S., Capp A., Dalley D., Goldstein D., Baber R., Friedlander M.L. (2005). Fertility- and menopause-related information needs of younger women with a diagnosis of early breast cancer. J. Clin. Oncol..

[56] Oosterhuis B.E., Goodwin T., Kiernan M., Hudson M.M., Dahl G.V. (2008). Concerns about infertility risks among pediatric oncology patients and their parents. Pediatr. Blood Cancer.

[57] Duffy C.M., Allen S.M., Clark M.A. (2005). Discussions regarding reproductive health for young women with breast cancer undergoing chemotherapy. J. Clin. Oncol..

[58] Schover L.R., Rybicki L.A., Martin B.A., Bringelsen K.A. (1999). Having children after cancer. A pilot survey of survivors’ attitudes and experiences. Cancer.

[59] Harris C.J., Rowell E.E., Corkum K.S. (2025). Special considerations in pediatric female fertility preservation. Semin. Pediatr. Surg..

[60] von Wolff M., Germeyer A., Böttcher B., Magaton I.M., Marcu I., Pape J., Sänger N., Nordhoff V., Roumet M., Weidlinger S. (2024). Evaluation of the Gonadotoxicity of Cancer Therapies to Improve Counseling of Patients About Fertility and Fertility Preservation Measures: Protocol for a Retrospective Systematic Data Analysis and a Prospective Cohort Study. JMIR Res. Protoc..

[61] Cohen C.B. (2009). Ethical issues regarding fertility preservation in adolescents and children. Pediatr. Blood Cancer.

[62] Feuerstein J.L., Menon S., Lockart B., Zarnegar-Lumley S., Johnson A.K. (2025). “I am so grateful you made me do this” Navigating AYAs-Caregiver Discordance on Shared Decision-making About Fertility Preservation. J. Pediatr. Adolesc. Gynecol..

[63] Boivin J., Pennings G. (2005). Parenthood should be regarded as a right. Arch. Dis. Child..

[64] Grundy R., Larcher V., Gosden R.G., Hewitt M., Leiper A., Spoudeas H.A., Walker D., Wallace W.H. (2001). Fertility preservation for children treated for cancer (2): Ethics of consent for gamete storage and experimentation. Arch. Dis. Child..

[65] Reiser E., Böttcher B., Ossig C., Schiller J., Tollinger S., Toth B. (2024). Female cancer survivors: Sexual function, psychological distress, and remaining fertility. J. Assist. Reprod. Genet..

[66] Quinn G.P., Murphy D., Knapp C., Stearsman D.K., Bradley-Klug K.L., Sawczyn K., Clayman M.L. (2011). Who decides? Decision making and fertility preservation in teens with cancer: A review of the literature. J. Adolesc. Health.

[67] Jadoul P., Dolmans M.M., Donnez J. (2010). Fertility preservation in girls during childhood: Is it feasible, efficient and safe and to whom should it be proposed?. Hum. Reprod. Update.

[68] Diesch T., von der Weid N.X., Szinnai G., Schaedelin S., De Geyter C., Rovó A. (2016). Fertility Preservation in Pediatric and Adolescent Cancer Patients in Switzerland: A Qualitative Cross-Sectional Survey. Cancer Epidemiol..

[69] Terenziani M., Spinelli M., Jankovic M., Bardi E., Hjorth L., Haupt R., Michel G., Byrne J. (2014). Practices of Pediatric Oncology and Hematology Providers Regarding Fertility Issues: A European Survey. Pediatr. Blood Cancer.

